# Mycobacteriophages: Windows into Tuberculosis

**DOI:** 10.1371/journal.ppat.1003953

**Published:** 2014-03-20

**Authors:** Graham F. Hatfull

**Affiliations:** Department of Biological Sciences, University of Pittsburgh, Pittsburgh, Pennsylvania, United States of America; University of North Carolina at Chapel Hill School of Medicine, United States of America

## What Are Mycobacteriophages?

Mycobacteriophages are viruses that infect mycobacterial hosts, such as *Mycobacterium tuberculosis* and *Mycobacterium smegmatis* [1]. Because the discovery and genomic characterization of mycobacteriophages has been the focus of integrated research and education programs, including the Phage Hunters Integrating Research and Education (PHIRE) and the Howard Hughes Medical Institute Science Education Alliance Phage Hunters Advancing Genomics and Evolutionary Science (HHMI SEA-PHAGES), thousands of phages have been isolated using a single host strain, *M. smegmatis* mc^2^155, over 500 of which have been completely sequenced [2]–[5]. These are mostly from environmental samples, but mycobacteriophages have also been isolated from stool samples of tuberculosis patients [6], although these have yet to be genomically analyzed. Clearly, these mycobacteriophages represent only a tiny piece of the overall phage population, which is predicted to include 10^31^ particles, making them the majority of all life-forms in the biosphere [7].

**Figure 1:**
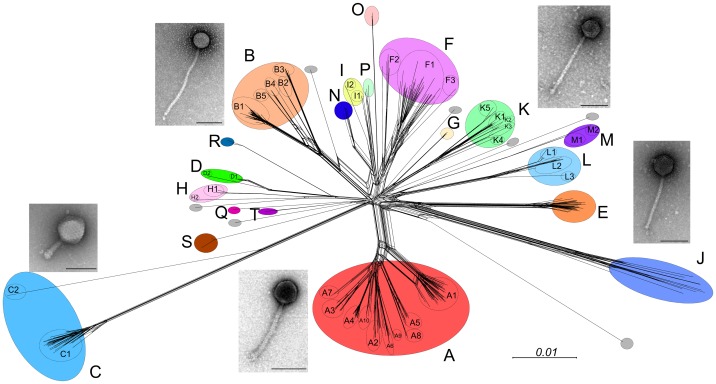
Diversity of mycobacteriophages. Sequenced genomes for 471 mycobacteriophages were compared according to their shared gene contents, and the relationships are displayed using Splitstree [24]. The genomes are clustered according to overall nucleotide sequence similarity, and the clusters (A, B, C…) correlate closely with their gene content. Colored circles encompass Clusters A–T as indicated, and grey circles represent singleton genomes that have no close relatives. Ten of the clusters are divided into subclusters (e.g., A1, A2, A3….) and are shown as circles within each cluster. Micrographs show the two morphotypes observed, typified by the myoviral Cluster C phages and the siphoviruses (all others) that primarily differ in tail length (scale bars, 100 nm). With the exception of one singleton (DS6A), all of the phages infect *M. smegmatis* mc^2^155. DS6A, the Cluster K phages and a subset of Cluster A phages also infect *M. tuberculosis*.

Mycobacteriophages display a remarkable genetic diversity (Figure 1). About 30 distinct types (called clusters, or singletons if they have no relatives) that share little or no nucleotide sequence similarity have been identified. Many of the clusters span sufficient diversity that the genomes warrant division into subclusters (Figure 1). However, the genomes are characteristically mosaic in their architecture [8], which is readily evident from the shared genes revealed by amino acid sequence comparisons [9]. There is also considerable range in overall guanine plus cytosine content (GC%), from 50.3% to 70%, with an average of 64% (*M. smegmatis* is 67.3%). Thus, phage GC% does not necessarily match that of its host, and the consequent mismatch of codon usage profiles does not appear to be detrimental. Because new mycobacteriophages lacking extensive DNA similarity with the extant collection are still being discovered, and as there are at least seven singletons for which no relatives have been isolated, we clearly have yet to saturate the diversity of this particular population. The pace of discovery is rapid, so the profile of diversity is constantly changing.

The collection of >50,000 genes can be sorted into >3,900 groups (phamilies) according to their shared amino acid sequences. Most of these phamilies (∼75%) do not have homologues outside of the mycobacteriophages and are of unknown function. Extrapolation to the broader population suggests that phages have the largest repertoire of unexplored sequences in nature. Genetic studies with mycobacteriophage Giles show that 45% of the genes are nonessential for lytic growth, raising questions as to how and why they were acquired [10].

## What Is the Basis of Viral Diversity?

If the diversity of phages of *M. smegmatis* mc^2^155 is this great, then the diversity of the phage population as a whole must be massive, as indicated from metagenomic studies [11]. Host range analysis shows that not all of these mycobacteriophages infect other strains of *M. smegmatis*, and only phages in Cluster K and in certain subclusters of Cluster A efficiently infect *M. tuberculosis* (Figure 1) [12]. However, mutants can be readily isolated from some phages that expand their host range to infect these other strains [12]. Thus, the observed phage diversity can be explained by assuming that wherever broad and diverse ranges of hosts are present, the phages can rapidly dance across the microbial landscape, using the hosts as “stepping-stones.” Migration across this landscape requires that the “stepping-stones” be spaced sufficiently close (genetically) to enable a host-range transition jump with either very few mutations or a gene acquisition event. The diversity of the mycobacteriophages therefore reflects the specific evolutionary pathways that they have pursued, sometimes occupying portions of the landscape dominated by strains other than *M. smegmatis* and probably outside of the genus *Mycobacterium*. For example, the lower GC% phages may have predominantly infected the lower GC% *Corynebacteria* and represent merely accidental tourists in the *Mycobacterium* locale. In this model, genes that were acquired because they were needed to grow in some recently visited host may not be needed in a current host but have yet to be selected against. Some of the nonessential Giles genes may thus correspond to these “legacy” genes. The host preference of the phages is presumably determined in part by the use of specific surface receptors, although few have been identified or characterized.

## How Can Mycobacteriophages Facilitate Tuberculosis Genetics?

*M. tuberculosis* is challenging to grow because of its slow growth rate (24-hour doubling time) and its pathogenicity. It was intractable to genetic manipulation until breakthroughs in the late 1980s that took advantage of mycobacteriophages to bootstrap methods for transfection and transformation, electroporation, plasmid vectors, and selectable markers [13]–[15]. Furthermore, phages have continued to be key players in developing a more facile genetic system. For example, several applications rely specifically on the ability of phages to inject their DNA into essentially every cell within a mycobacterial population, making phages ideal for transposon delivery and preparation of complex transposon libraries [16], gene replacement using specialized transduction [17], and tuberculosis (TB) diagnosis by inclusion of a reporter gene [18], [19]. But the component parts of the phages also have tremendous utility and often work in *M. tuberculosis* even if the phage doesn't actually infect it. Examples include integration-proficient plasmid vectors and recombineering strategies, although there are numerous other potential applications that have yet to be exploited. The overall diversity of the phages massively fuels these approaches, providing a toolkit of over 50,000 genes that can be exploited.

## Do Mycobacteriophages Influence Mycobacterial Physiology?

It is well established that phage-encoded toxins contribute to the virulence of a variety of bacterial pathogens, including *Escherichia coli*, *Salmonella* sp., *Coynebacterium diphtheria,* and *Vibrio cholera*. *M. tuberculosis,* however, clearly differs from these in its pathogenesis, and there is no evidence for phage-encoded toxins. Most *M. tuberculosis* strains carry one or both of two small (∼10 kbp) prophage-like elements φRv1 and φRv2, but it seems unlikely they contribute to virulence. Some mycobacteriophages (in Cluster D) do encode a vegetative insecticidal protein (VIP2)-like insect toxin genes that could confer virulence to a bacterial host, although it is unclear what that host might be, or what it might infect; they do not infect *M. tuberculosis*. We note that several mycobacterial strains, such as *M. cannetti*, *M. marinum*, *M. abscessus*, and *M. ulcerans,* carry seemingly intact prophages, which could influence their biology.

Expression of phage-encoded proteins is only one way that phages can influence their hosts. An alternative route is by integration of the phage genome into a host gene that is required for some physiological process. Phage integration is typically site-specific—involving integrase-mediated recombination between phage and bacterial attachment sites (*attP* and *attB*, respectively)—and two distinct types of enzymes are used. Tyrosine-integrases are the most common and typically mediate integration into a host tRNA gene (the well-studied phage lambda integrase is a notable exception). Because such phages carry the 3′ half of the tRNA gene at *attP*, tRNA functionality is maintained following prophage establishment. In contrast, phages using a serine-integrase typically use an *attB* site located within a host's protein-coding genes, which is interrupted by the integration event. These *attB* loci are small and cannot be readily predicted bioinformatically, and relatively few have been identified experimentally. We predict there are six to 12 different *attB* sites within *M. smegmatis* and *M. tuberculosis,* and integration into those that are mediated by a serine-integrase could potentially alter host physiology.

For mycobacteriophage Bxb1, the consequences of integration for host physiology are well established [20]. Bxb1 uses a serine-integrase to integrate into an *attB* site located within the *groEL1* gene of *M. smegmatis* [21]. GroEL1 serves as a dedicated chaperone for regulation of mycolic acid biosynthesis and is required for the formation of mature biofilms. Thus, lysogens of Bxb1 are defective in forming biofilms, perhaps providing a selective advantage as cheaters within a broader population of non-lysogenic biofilms [22]. This phenotype would be easy to miss unless searching for it specifically, and we predict that many other phages that use serine-integrases also influence host physiology.

## Do Mycobacteriophages Have Therapeutic Potential?

The increased prevalence of antibiotic resistance in bacterial pathogens has spurred renewed interest in the therapeutic use of bacteriophages. Although phages have been extensively used therapeutically in former Soviet Union countries, they have yet to find widespread use in either the United States or in Europe. Phage preparations have been approved for use against *E. coli* and *Listeria* meat contamination, and trials are in progress for control of several human infections. Skin afflictions and burns seem to be especially attractive targets.

What about phage therapy for tuberculosis? Antibiotic resistance is certainly a growing and worrisome development, particularly with the emergence of extensively drug-resistant (XDR) and totally drug-resistant (TDR) strains, both of which are especially difficult to control. Delivery of phages to the lungs should be relatively simple, although there is considerable doubt as to whether they would effectively reach their bacterial hosts, which may be intracellular and within granulomas. An intriguing suggestion for addressing the access question is to use infected surrogate mycobacterial cells for the delivery [23]. Unfortunately, relatively few efficient phage killers of *M. tuberculosis* are available, and because phage resistance is to be expected, a suite of three to six phages that efficiently kill *M. tuberculosis* and elicit different resistance mechanisms in the host are needed. Because only a subset of those phages isolated on *M. smegmatis* also infect *M. tuberculosis*, isolation of additional phages known to infect *M. tuberculosis* is desirable.

In spite of these concerns, there is considerable potential to use phages prophylactically by interfering specifically with TB transmission. For example, if a patient was diagnosed with tuberculosis, family members and coworkers could aspirate phages into the upper respiratory tract, where the phages could infect and kill *M. tuberculosis* cells as they are breathed in and before they establish an infection. As transmission typically involves small numbers of cells, it ought to be possible to deliver a sufficient amount of phage particles while minimizing phage-resistance. Safety is not expected to be a concern, and there should be few impediments to evaluating mycobacteriophages prophylaxis.
